# Knowledge, provision of information and barriers to high quality medication abortion provision by pharmacists in Uttar Pradesh, India

**DOI:** 10.1186/s12913-019-4318-4

**Published:** 2019-07-11

**Authors:** Nadia Diamond-Smith, Joanna Percher, Malvika Saxena, Pravesh Dwivedi, Aradhana Srivastava

**Affiliations:** 1 Department of Epidemiology and Biostatistics and Institute for Global Health Sciences, University of California, San Francisco, 550 16th Street, 3rd Floor, San Francisco, CA 94158 USA; 2 University of California, Berkeley, USA; 3 Public Health Foundation, Gurugram, India; 4 Lucknow University, Lucknow, India

**Keywords:** Medication abortion, India, Quality of care, Mixed-methods

## Abstract

**Background:**

The use of medication abortion is increasing rapidly in India, the majority of which is purchased through pharmacies. More information is needed about the quality of services provided by pharmacist about medication abortion, especially barriers to providing high quality information. The goal of this study was to explore the quality of pharmacist medication abortion provision using mixed methods to inform the developed of an intervention for this population.

**Methods:**

Data was collected via convenience sampling using three methods: a quantitative survey of pharmacists (*N* = 283), mystery clients (*N* = 111), and in-depth qualitative interviews with pharmacist (*N* = 11). Quality indictors from the quantitative data from surveys and mystery clients were compared. Qualitative interviews were used to elucidate reasons behind findings from the quantitative survey.

**Results:**

Quality of information provided to client purchasing medication abortion was low, especially related to timing and dosing of misoprostol (18% of pharmacists knew correct timing) and side effects (31% not telling any information on side effects). Mystery clients reported lower quality (less correct information) than pharmacists reported about their own behaviors. Qualitative interviews suggested that many barriers exist for pharmacists, including perceptions about what information clients can understand and desire, and also lack of comfort giving certain information to certain types of clients (young women).

**Conclusions:**

It is essential to improve the quality of information given to client purchasing medication abortion from pharmacists. Our findings highlight specific gaps in knowledge and reasons for poor quality information. Differences in guidelines available at that time from the Indian Government, World Health Organization, and the medication abortion boxes may lead to confusion amongst pharmacists and potentially clients. Interventions need to improve both knowledge about medication abortion and also biases in the provision of care.

**Electronic supplementary material:**

The online version of this article (10.1186/s12913-019-4318-4) contains supplementary material, which is available to authorized users.

## Background

Although abortion was legalized in India through the Medical Termination of Pregnancy (MTP) act in 1971, access to and knowledge of safe abortion services in public clinics remains limited [1–6]. While the MTP act requires a medical prescription to obtain medication abortion (MA) drugs at a service delivery point (hospital, clinic or pharmacy), many clients go directly to pharmacies, and pharmacists and/or pharmacy workers become their first point of care [2, 7–12]. It is currently estimated that 73% of the 15.6 million annual abortions in India are induced through MA outside of facilities, with an estimated 87% of these abortions through combination packs in which mifepristone and misoprostol are packaged together [13]. Thus, pharmacy workers are often the primary means of communication about how to take MA, what to expect, and when to seek care, to clients purchasing MA [2, 7, 8, 11].

Medication abortion using mifepristone and misoprostol has been shown to be safe and effective when used appropriately, both in and out of the clinic setting [14, 15]. While 14% of the 56 million abortions globally are classified as “least safe,” done by untrained providers with dangerous methods, increasing self-use of MA is credited with helping drive down abortion-related morbidity and mortality [16–18]. Much out-of-facility use is in places where abortion is legally restricted but misoprostol is available due to its registration for other health concerns, often gastric ulcers [19]. In India, though abortion is legal, barriers to clinic-based care and the ease and relative low cost of accessing out-of-facility MA in many states has spurred its high use. While MA is safe, individuals taking the medication need to have sufficient information to have a satisfactory, supported experience. In Uttar Pradesh, where an estimated 3.15 million abortions take place annually, over 1 million individuals are treated for induced abortion complications at clinics [20]. Many of these are likely unnecessary interventions because individuals did not know how to differentiate between normal progress of MA versus a complication necessitating additional care. Furthermore, treatment for these perceived complications often use dilation and curettage, an invasive and outdated procedure, used at a higher rate in Uttar Pradesh than in other Indian states studied by Guttmacher in 2015 [21].

The reliance on pharmacies for the provision of MA in India has been known for some time, but it is less well studied. The extant research points to low quality of knowledge on MA among pharmacists [2, 7, 8, 10–12]. In a 2015 study in Madhya Pradesh, only 35% of pharmacists provided correct information on MA regimens (including dosage and timing), and 28% correctly advised clients on where to go should complications arise [8]. A study in the states of Gujarat and Jharkhand found that 23% of pharmacists interviewed gave no information to clients when selling MA, and only 15% provided information on side effects [2]. Other studies in Delhi, Bihar, and Gujarat have found similar results. Post-abortion family planning was rarely offered or discussed at the time of dispensing MA medication [12]. These findings suggest poor quality of care for MA services through pharmacies.

In order to measure the quality of MA information and care provided by pharmacists, it is essential to understand what the published MA regimens that would potentially be available to pharmacists in this setting at the time of data collection. One challenge for MA quality provision is that multiple and differing guidelines exist in India. To illustrate this, we show the World Health Organization (WHO), the Indian Handbook on Medical Methods of Abortion and the instructions from inserts in three popular MA combination packs in India (Unwanted, MMkit and TermiPill) [22, 23] (Table 1). Of note, the WHO MA guidelines are for up to 63 days gestation and the Indian Handbook on Medical Methods of Abortion has not been updated since the gestational limit for MA was extended from 49 to 63 days in the private sector. While all have the same dose and route of administration for mifepristone, it is important to note that they differ slightly in the timing between mifepristone and misoprostol, with the WHO recommending 1–2 days (24–48 h), the Indian Handbook between “day 1 and day 3”, and the MA kits 1–3 days later. The Indian Handbook on Medical Methods of Abortion also differs in that it recommends only 400 mcg, or two pills, of misoprostol, rather than the 800 mcg recommended by WHO and in the instructions in the MA kits. Route of administration of misoprostol also differs by source.

**Table 1:** Instructions for dosage, timing, and route of administration from three sources

Category	WHO guidelines for MA use up to 63 days	Indian Handbook on Medical Methods of Abortion (Ministry of Health and Family Welfare, Government of India, 2016)	Instruction inserts from three MA combination pack kits (Unwanted, MM kit, and TermiPill brands)
Timing of mifepristone and misoprostol	Day 1: mifepristone 1–2 days later (24–48 h) misoprostol	Day 1: mifepristone Day 3: misoprostol	Day 1: mifepristone 1–3 days later misoprostol
Dose of mifepristone	200 mg	200 mg	200 mg
Route of mifepristone administration	Oral	Oral	Oral
Dose of misoprostol	400 mcg (2 pills) if taken orally up to 7 weeks 800 mcg (4 pills) if up to 9 weeks taken vaginally, buccally, or sublingually	400 mcg (2 pills) at same time	800 mcg (4 pills) at same time
Route of misoprostol administration	Up to 7 weeks: Vaginal, buccal, sublingual or oral Up to 9 weeks: Vaginal, buccal, or sublingual	Sublingual: most recommended Buccal: recommended Vaginal: recommended Oral least recommended but still OK	Vaginal

Note: Since the time of data collection, some of these guidelines have been changed, however, we present the guidelines available at the time of data collection to more accurately be able to compare the knowledge and quality of information provided by pharmacists at that time to resources available to them

Quality of care, including knowledge, practices, and behaviors, is notoriously challenging to measure for many health services, including reproductive health services such as family planning and MA. The standard methods, including provider self-report, client exit interviews, and observations, all suffer from biases [24]. Mystery clients have been found to provide additional information about quality of care for family planning, and it has been argued that this approach is especially valuable combined with provider self-report [24, 25]. However, few studies to date have utilized mystery clients to understand quality for MA provision through pharmacies in India (or other outlets), but are likely to provide an additional perspective on the quality of information and care provided to clients seeking MA [8, 10, 18, 26–30].

## Methods

This paper aims to explore the knowledge and information given by pharmacists who sell MA, and the barriers to providing high quality information and care through mixed methods, including a survey, mystery clients, and in-depth interviews with pharmacists. The survey was designed to obtain a larger sample of pharmacists’ self reports of their knowledge and practices. Mystery clients were used to see how these self-reports translate into behaviors towards clients. Qualitative interviews were used to explore pharmacist’s knowledge and barriers to providing quality care in depth.

### Study setting

The study was conducted in the urban and peri-urban areas of three districts in Uttar Pradesh – Lucknow, Kanpur Nagar and Unnao. The districts were selected on the basis of an ongoing study on maternal health quality, of which this was a sub-study. In these districts are located Lucknow and Kanpur - two of the largest cities in Uttar Pradesh, with a significant urban population and a large number of pharmacies. The social marketing firm that we partnered with estimated that there were 3,000–4,000 pharmacies in these three districts. We therefore expected larger clientele of MA in such settings. The third district, Unnao, was selected to represent a more peri-urban population. Data was collected between November 2017–March 2018. This study received Human Subjects Approval from the Public Health Foundation of India, India and the University of California, San Francisco.

### Methods and data collection

In-depth interviews were conducted with eleven pharmacists who had sold MA to clients. Pharmacists providing MA services were identified in collaboration with a social marketing firm that also supplies MA kits to pharmacists. The social marketing firm had a list of pharmacists who they had provided MA kits to, which they shared with us, allowing us to approach pharmacists who we knew were providing MA, using convenience sampling from this list. Verbal informed consent was obtained before the interview and also for recording. Notes were taken in the event the provider refused to be recorded. An information sheet with the investigators’ contact details was shared with all respondents, and they were encouraged to call in case they had any doubts later on. Interviews covered pharmacists’ experiences with and barriers to providing MA (Additional file 1). Interviews were conducted in Hindi by Indian research assistants and translated to English. Interviews were thematically analyzed after coding by a team of 4 researchers (all authors) with a Grounded Theory Approach using AtlasTi software [31].

We then conducted a survey with pharmacists– 176 randomly selected (using simple random sampling) from the roster of a social marketing organization, and a random sample of 119 pharmacies not on the social marketing firm’s roster, from the same areas. The only criterion was that they had to be located in urban or peri-urban areas. The investigators then approached the pharmacy workers and interviewed upon consent. A total of 295 pharmacists were surveyed, with 283 completing the full survey. A team of six public health researchers conducted the survey, following 2 days of training. The survey was conducted in Hindi after obtaining verbal informed consent. The informed consent also included consent for mystery clients to visit the facility at some point in the next 6 months. Data was collected digitally on tablets using the Survey CTO software. The questionnaire comprised of questions to assess pharmacist knowledge and practices surrounding medication abortion, along with a few demographic questions. Each survey was about 30 min long. Confidentiality of the respondent was strictly maintained as per protocol to prevent any leakage of identity or information disclosed. The place and time of interview was decided by the respondent and only conducted as per their comfort. Pharmacists were also visited during non-peak times to reduce the number of potential customers they might encounter during the interview. Interview was paused if a customer arrived, and resumed when she or he had left. Any doubts that the respondent had were satisfied before commencing with the interview.

Mystery clients visited 111 of the pharmacies that were surveyed (not those on the social marketing firm’s roster) about 2 months after the initial survey. Research assistants were deployed as mystery clients assuming four different profiles: a younger, unmarried woman (~ 18 years), a younger unmarried man (~ 20 years), a married woman (~ 28 years) and a married man (~ 30 years). The research assistants were recruited from similar communities where the study took place. Mystery clients completed a three-day training with pilot testing in the field and in-depth role plays. Mystery clients approached each pharmacist by saying that they had an unplanned pregnancy, and were trained to only probe on specifics (MA itself, side effects, dose, etc.) if the pharmacist did not willingly volunteer that information. Immediately after each interaction, the mystery client filled out a standardized digital survey about their experience, which included an open-ended section for additional notes.

Descriptive statistics were calculated for survey data (pharmacist survey and mystery client survey). Pharmacist and mystery client responses are compared where possible. All survey analysis was conducted using Stata 15MP [32]. Quality was assessed in comparison to guidelines presented in Table 1.

## Results

All pharmacists in the interviews were males and had either completed a diploma or degree course in pharmacy. As can be seen in Table 1, 99.3% of the survey respondents were men, and most were graduates in something other than pharmacy (55.5%), with about 18% having a degree or diploma in pharmacy. While it is mandatory to have a degree in pharmacology to open a pharmacy shop, in reality the person working day-to-day behind the counter may not be trained in pharmacy. We interviewed whoever was at the pharmacy seeing clients on the day of the visit as this most accurately represented who clients would be interacting with.

### Pharmacist’s initial interactions with clients

Twenty-four percent of pharmacists said that none of the people they had sold MA to came with a prescription (Table 2). Very few (5.4%) of the mystery clients were asked if they had a prescription. Only 5% of pharmacists reported that they did not determine the woman’s gestational age in any way, and of those that did, the majority (57.4%) did so by trusting what the woman told them, 66.7% did it based on the last menstrual period (LMP), and 24.4% based on a doctor’s report. The majority (76.6%) of mystery clients were asked their last menstrual period, and 87.4% were asked if they took a pregnancy test.

**Table 2:** Profile of Pharmacists in the survey (*N* = 283)

	N (%)
Gender	
Male	281 (99.3)
Female	2 (0.7)
Degree	
M. Pharma / B. Pharma	24 (8.5)
Diploma in pharma	28 (9.9)
Post graduate-others	35 (12.4)
Graduate-others	157 (55.5)
Not a graduate	37 (13.1)
Others	2 (0.7)
Percentage of people who came to the pharmacy for MA in past month with a doctor’s prescription	
0%	68 (24)
1–25%	114 (40.3)
26–50%	53 (18.7)
51–75%	20 (7.1)
Over 75%	28 (9.9)
Total	283 (100)

### Knowledge of and information sharing on MA dosing and timing

In the depth-interviews, all pharmacists reported that they explained the same dose as in the kit and recommended by the WHO guidelines (Table 1). However, only 38.2% of pharmacists stated that dosage in the quantitative survey (Table 3). Five pharmacists (1.8%) followed the India guidelines by advising only 400 mcg misoprostol initially be taken, followed with an additional 400 mcg “if needed,” with three explicitly stating to take the second dose only if bleeding does not occur after 24 h. In the survey, 73% of pharmacists reported spacing between mifepristone and misoprostol somewhere in the range of 24–48 h, and 7% reported that they did not know the spacing (Table 3). Just over half (56%) of mystery clients were told a timing between the two medications of 12–24 h.

**Table 3:** Medication abortion counseling: comparing pharmacist survey and Mystery Clients

	Pharmacist Survey *N* = 283 N (%)	Mystery Client *N* = 111 N (%)
Determines gestational age, and if so, how?		
Does not determine the estational age	13 (4.59)	No info
Trust on client saying	155 (57.41)	No info
Based on last menstrual period	180 (66.67)	85 (76.58)
As per doctor assessment/report	66 (24.44)	No info
Other	1 (0.37)	No info
Asked if she had taken a pregnancy test		97 (87.39)
Tell clients to have anywhere between a 24–48 h gap between mifepristone and misoprostol	207 (73.14)	60 (55.56)
Don’t know	21 (7.42)	No info
Reported taking all 4 misoprostol tablets together (800 mcg total)	51 (18.02)	No info
Don’t know	21 (7.42)	No info
Reported taking misoprostol tablets in two doses of 2 pills (800 mcg total)	109 (38.51)	No info
Route to take misoprostol		
Oral	256 (90.46)	99 (89.19)
Vaginal	114 (40.28)	12 (10.81)
Sublingual	20 (7.1)	3 (2.70)
Buccal	9 (3.2)	3 (2.70)
Other	4 (1.4)	0 (0)
Don’t know/Told nothing	11 (3.9)	4 (3.60)
Potential side effects/reactions to MA told to clients		
Does not tell MA clients about possible reactions (side effects) to MA	88 (31.1)	15 (13.51)
Nausea/vomiting	65 (33.3)	9 (8.11)
Headache	31 (15.9)	1 (0.90)
Diarrhea	4 (2.1)	
Lower abdomen pain	72 (36.92)	62 (55.86)
Heavy bleeding	169 (86.67)	43 (38.74)
Dizziness/weakness	37 (19)	29 (26.13)
Fever	12 (6.15)	
Other	41 (21)	3 (2.70)

The WHO guidelines and MA kit instructions both advise for four pills, 800 mcg total, of misoprostol to be taken together, however only 18% of pharmacists advised for the 4 misoprostol pills in the combination pack to be taken together (with another 7% reporting that they did not know). About a third (38.5%) of pharmacists advised the client to take the 4 pills of misoprostol in two doses of 400 mcg each (two pills), and 7.1% advised it to be taken in 3 or four doses. In the interview data, pharmacists reported telling clients to have a 12 to 24 h gap between two doses of two pills of misoprostol.

*I explain to them to take the big tablet with water orally after dinner, and then two of the small tablets after 24 hours gap and the remaining two tablets the next day in the same manner. (Pharmacist 10, age 30–34 years)*

The majority of pharmacists told women to take the misoprostol tablets orally (90.5%), with 40.3% and/or reporting that they tell women to take it vaginally. While vaginal administration is recommended by all three regimens, oral is only recommended until 49 days gestation in the WHO guidelines, is the “least recommended” in the India guidelines, and is not presented in the MA kit instructions. Very few reported telling women to take the misoprostol sublingually or buccally (respectively) or did not know. From the mystery client data, almost 90% were told to take the misoprostol orally, 11% vaginally, and about 3% buccally and sublingually each.

Some pharmacists described telling clients to take MA orally because they felt that it would scare the clients and that it was an overly “complicated” method. As one pharmacist described, he felt it would be difficult for the clients to understand how to take it vaginally:

*R: I don’t suggest inserting pills vaginally because this route may cause excessive bleeding but yes I suggest this method when the duration of pregnancy is long.*

*I: Do you explain to clients about other options for taking the second dose of medication?*

*R: Yes, I do explain how else to take the second dose. I explain them about sublingual method; tell them to keep two tablets below tongue. But sometimes when they don’t understand I just ask them to take orally with water. (Pharmacist 6, age 40–44 years)*

A number of pharmacists said that it would make them uncomfortable or be awkward to tell women about the vaginal method—both for them and the woman.

*I don’t suggest this mode of administration as it may be awkward for both of us. I don’t know whether they would understand or not, I never tried to. I just ask them to take orally. (Pharmacist 4, age 40-44 years)*

### Information about side effects

Almost a third (31.1%) of pharmacists in the survey reported not telling clients about side effects, and of those that did, the most commonly mentioned were bleeding (86.7%), lower abdomen pain (36.9%), nausea/vomiting (33.3%) and dizziness (19%) (Table 3). Only 13% of the mystery clients were not told about side effects, however, bleeding was mentioned less frequently (38.7%), pain more frequently (55.9%) and nausea/vomiting less frequently (8.1%); other side effects were mentioned at similar frequencies.

Qualitatively, pharmacists had mixed feelings about telling clients about side effects. One pharmacist clearly explained that he did not tell clients about side effects because this would frighten them and make them not want to take (or buy) the kit.

*I just tell them there is no side effect as I do not want to talk more about it….If I tell them about the progression of abortion then they will not take the kit! If I tell them that the first dose kills the foetus and the subsequent doses expel the remains, they will not take the pills, as it might be scary for them. So I don’t tell them anything. (Pharmacist 2, age 35–39 years)*

On the other hand, many pharmacists wanted to provide information and felt strongly that clients needed more information,

*For quality care clients must know about the side effects of taking MA pills because I am seeing nowadays that mostly teenagers are using it and they do not know anything about the side effects or progression of abortion like excessive bleeding, cramps etc. – (Pharmacist 7, age 40–44 years)*

However, some pharmacists provided non-evidence based information about dosing/timing and side effects, for example avoiding cold foods for 3–4 days after taking MA, or taking the tablets with milk.

### Barriers to providing high quality care: fears, misperceptions and stigma

When asked about barriers to providing high quality care, pharmacists cited business-related concerns. For example one pharmacist explained how he did not have the time to explain detailed information to each client, as doing so would limit the number of customers he would be able to serve:

*“For me the main barrier is time constraint. I can’t give more than 5 minutes to one client because I will lose 2-3 clients while dealing with one client and this is not good for my business” (Pharmacist 7, age 40-44 years).*

When asked what they told women or men who came with unintended pregnancies, 52% of pharmacists said they told clients to continue with the pregnancy if she had none or only one child. In the interviews, some pharmacists felt that it was difficult to provide information to certain types of respondents, mainly unmarried women, about pregnancy or their need for MA, because they did not feel comfortable questioning them directly about these topics. Many pharmacists openly discussed this being a barrier in their caregiving. A few pharmacists clearly stated that they felt “awkward” explaining to women about how to take MA and others, such as the one below, said that they felt more comfortable talking with male clients than female:

*It doesn’t matter to me whether the client is a male or female, I just do my job but yes I can say that it’s easy to deal with male clients as I can ask them directly. In case of female clients I have to be extra cautious and sensitive before asking any personal questions like date of last menstrual period or pregnancy test etc. as they may feel uncomfortable in sharing these details with a male. (Pharmacist 7, age 40–44 years)*

One pharmacist summed up many of the barriers discussed above clearly, relating them to the overall cultural and social norms

“*Frankly speaking we as a society are still… shy to talk about these problem.”(Pharmacist 8, age 35–39 years)*

## Discussion

Information on correct MA timings, route of administration and dosage, normal progression, side effects/complications, and appropriate referral are some of the critical elements in ensuring safe medication abortion. Our study revealed that quality of knowledge about MA is mixed among pharmacists in Uttar Pradesh—supporting past evidence in India (2,7,8,10–12.) Similar gaps in pharmacist knowledge on MA have been identified in other settings where individuals may purchase MA over the counter, including Kenya, Mexico, and the Dominican Republic [18, 26–28]. However, knowledge levels did not translate into behaviors, as we identified clear components of MA use for which pharmacists are providing incorrect information, most especially related to misoprostol timing and dose. One explanation of this may be that providers are confused about appropriate guidelines and thus nervous about what to tell clients, leading them to provide incorrect information. A careful analysis of the regimens pharmacists describe compared to the differing guidelines provided by WHO, Indian Handbook, and MA kit instructions, highlight the complexities of interpreting the “quality” of pharmacists knowledge. For example, the recommendations differ in exactly how long to wait between the mifepristone and misoprostol, and, depending on one’s interpretation of “1–2 days” (does this mean 24–48 h?), it would be possible to advise varying gaps and still be within a certain interpretation of the recommendations. Given the varying recommendations in the guidelines, it may be challenging for pharmacists to know what to tell clients, and clients to know what to take and how to take it.

The mixed methods approach used in this study provides additional insights into the quality of care provided to MA clients, aside from pharmacist knowledge itself. Overall, mystery clients reported lower quality information than was self-reported by the pharmacists, similar to another study on MA that employed both a survey and mystery clients in Madhya Pradesh [8]. This suggests that there may be a gap between knowledge and practices, which could be due to time constraints when actually counseling or lack of comfort counseling about MA, potentially to specific types of clients. The pharmacists, who were all male, discussed in the in-depth interviews being uncomfortable talking to female clients, especially about using the vaginal route of administration of misoprostol. This sheds light on the survey findings that many more pharmacists recommended the oral route than guidelines would suggest. This finding that providers are giving differential information to different types of clients, in addition to a general know-do gap, adds to the existing literature and informs potential focuses for interventions. Currently, most pharmacists in India are male, as is true for most of the formal labor force in this setting where women’s empowerment and formal labor force participation is low.

Addressing provider biases through interventions that help pharmacist become comfortable communicating outside of the norms of society could help reduce stigma. Another approach could be recruiting and training more female pharmacists. Additionally, engaging pharmacists who do not provide information for fear of losing business (due to lost time or fear of clients being scared) is essential; the same is true for clinicians who could also view MA as taking away business opportunities. Bringing creative approaches used in other countries to UP to incentivize pharmacists and providers, such as financial incentives for providing counseling or providing informational materials which clients can read/view on their own or even take home with them, could remedy these issues. Successful related interventions include a project in Peru involving pharmacist training, dispersal by pharmacists of “STD/HIV prevention packs” with information to clients, and the creation of a referral system to clinics for STI management [33]. Training pharmacists on MA using a harm reduction framework has also demonstrated successful abortion and quality of care outcomes [34]. While training at scale for pharmacists and pharmacy workers throughout India may be difficult to implement and sustain, there is the need for further innovation to improve the information provision and quality of care in this context. Interventions that bypass pharmacists as the provider of information, such as hotlines or other means of support, are another approach for improving the quality of information that women receive.

This study has several limitations. In-depth interviews may not be generalizable to other pharmacists even in the three districts from which data was collected. Additionally, interviews were collected about 1 year before the survey, and thus, it is possible that knowledge or practices regarding MA changed between those time periods. Quality measures were not exactly the same in the pharmacist and mystery client surveys and therefore we are not able to compare every measure. Most of the pharmacists in our sample were male, so we know do not know whether differences exist between male and female pharmacists; however, very few pharmacist in this setting are female, therefore our data is reflective of most pharmacists in this setting. Finally, since pharmacists gave consent for mystery client visits and knew a mystery client might visit, it is possible that they were behaving differently than they would have otherwise. However, there was a 2–3 month time gap between the survey and mystery client visit, and no mystery client interactions gave any indication that pharmacists were suspicious. Despite these limitations, this study has many strengths, including the mixed methods approach applied to an understudied topic of growing importance.

## Conclusions

Gaps remain between knowledge and practices surrounding MA provision through pharmacies in Uttar Pradesh, India. Lack of consistency in recommendations about MA in India is potentially feeding into poor or mixed quality of information being provided to clients purchasing MA. It is critical that the multiple stakeholders involved in assuring the quality of MA provision in India come to a consensus about recommendations so pharmacists providing MA, clients (regardless of gender) purchasing MA, those taking MA, and other providers and support for individuals taking MA, can be clear on safe and effective MA regimens. MA is a relatively small share of product sales for pharmacists, therefore, innovative approaches that encourage them to learn and provide appropriate information to all clients is needed. Above and beyond the translation of knowledge into practice, it is clear that pharmacists are providing poorer quality care to female clients, due to cultural and gender norms influencing communication about sensitive issues between the sexes. This is a worrisome finding that must be addressed to ensure women are able to take MA with accurate and complete information. Despite misinformation and socio-cultural barriers, many pharmacists are trying to and want to provide appropriate information to clients purchasing MA, suggesting that there is an opportunity to improve the quality of care for MA in this setting.

## Additional file

Additional file 1: Interview with Chemist and Clinical MA Providers/chemists. (DOCX 123 kb)

## Data Availability

The datasets used and/or analysed during the current study are available from the corresponding author on reasonable request.

## Abbreviations

LMP: Last menstrual period
MA: Medication Abortion
MTP: Medical Termination of Pregnancy
STD/HIV: Sexually Transmitted Diseases/Human Immunodeficiency Virus
STIs: Sexually Transmitted Infections
WHO: World Health Organization
