# Supplementary material for: Knowledge, provision of information and barriers to high quality medication abortion provision by pharmacists in Uttar Pradesh, India
Source: BMC Health Serv Res. 2019 Jul 11;19:476. doi: 10.1186/s12913-019-4318-4 (PMC6622002; doi:10.1186/s12913-019-4318-4)
Supplement: Supplementary file 1 — Interview with Chemist and Clinical MA Providers/chemists. (DOCX 123 kb) [file 12913_2019_4318_MOESM1_ESM.docx]

Table 1: Instructions for dosage, timing, and route of administration from three sources

| **Category** | **WHO guidelines for MA use up to 63 days** | **Indian Handbook on Medical Methods of Abortion** (Ministry of Health and Family Welfare, Government of India, 2016) | **Instruction inserts from three MA combination pack kits (Unwanted, MM kit, and TermiPill brands)** |
| --- | --- | --- | --- |
| Timing of mifepristone and misoprostol | Day 1: mifepristone  1-2 days later (24-48 hours) misoprostol | Day 1: mifepristone  Day 3: misoprostol | Day 1: mifepristone  1-3 days later misoprostol |
| Dose of mifepristone | 200 mg | 200 mg | 200 mg |
| Route of mifepristone administration | Oral | Oral | Oral |
| Dose of misoprostol | 400 mcg (2 pills) if taken orally up to 7 weeks  800 mcg (4 pills) if up to 9 weeks taken vaginally, buccally, or sublingually | 400 mcg (2 pills) at same time | 800 mcg (4 pills) 800 mcg (4 pills) at same time |
| Route of misoprostol administration | Up to 7 weeks: Vaginal, buccal, sublingual or oral  Up to 9 weeks: Vaginal, buccal, or sublingual | Sublingual: most recommended  Buccal: recommended  Vaginal: recommended  Oral least recommended but still OK | Vaginal |

*Since the time of data collection, some of these guidelines have been changed, however, we present the guidelines available at the time of data collection to more accurately be able to compare the knowledge and quality of information provided by pharmacists at that time to resources available to them.

Table 2: Profile of Pharmacists in the survey (N=283)

|  | **N (%)** |
| --- | --- |
| **Gender** |  |
| Male | 281 (99.3) |
| Female | 2 (0.7) |
| **Degree** |  |
| M. Pharma / B. Pharma | 24 (8.5) |
| Diploma in pharma | 28 (9.9) |
| Post graduate-others | 35 (12.4) |
| Graduate-others | 157 (55.5) |
| Not a graduate | 37 (13.1) |
| Others | 2 (0.7) |
| **Percentage of people who came to the pharmacy for MA in past month with a doctor’s prescription** |  |
| 0% | 68 (24) |
| 1-25% | 114 (40.3) |
| 26-50% | 53 (18.7) |
| 51-75% | 20 (7.1) |
| Over 75% | 28 (9.9) |
| Total | 283 (100) |

Table 3: Medication abortion counseling: comparing pharmacist survey and Mystery Clients

|  | Pharmacist Survey N=283 N (%) | Mystery Client N=111  N (%) |
| --- | --- | --- |
| **Determines gestational age, and if so, how?** |  |  |
| Does not determine the gestational age | 13 (4.59) | No info |
| Trust on client saying | 155 (57.41) | No info |
| Based on last menstrual period | 180 (66.67) | 85 (76.58) |
| As per doctor assessment/report | 66 (24.44) | No info |
| Other | 1 (0.37) | No info |
| **Asked if she had taken a pregnancy test** |  | 97 (87.39) |
| **Tell clients to have anywhere between a 24-48 hour gap between mifepristone** **and misoprostol** | 207 (73.14) | 60 (55.56) |
| Don't know | 21 (7.42) | No info |
| **Reported taking all 4 misoprostol tablets together (800 mcg total)** | 51 (18.02) | No info |
| Don't know | 21 (7.42) | No info |
| **Reported taking misoprostol tablets in two doses of 2 pills (800 mcg total)** | 109 (38.51) | No info |
| **Route to take misoprostol** |  |  |
| Oral | 256 (90.46) | 99 (89.19) |
| Vaginal | 114 (40.28) | 12 (10.81) |
| Sublingual | 20 (7.1) | 3 (2.70) |
| Buccal | 9 (3.2) | 3 (2.70) |
| Other | 4 (1.4) | 0 (0) |
| Don't know/Told nothing | 11 (3.9) | 4 (3.60) |
| **Potential side effects/reactions to MA told to clients** |  |  |
| Does not tell MA clients about possible reactions (side effects) to MA | 88 (31.1) | 15 (13.51) |
| Nausea/vomiting | 65 (33.3) | 9 (8.11) |
| Headache | 31 (15.9) | 1 (0.90) |
| Diarrhea | 4 (2.1) |  |
| Lower abdomen pain | 72 (36.92) | 62 (55.86) |
| Heavy bleeding | 169 (86.67) | 43 (38.74) |
| Dizziness/weakness | 37 (19) | 29 (26.13) |
| Fever | 12 (6.15) |  |
| Other | 41 (21) | 3 (2.70) |
